# Duties of Dentist to Patient—Duties of Patient to Dentist

**Published:** 1895-10

**Authors:** A. W. M'Candless

**Affiliations:** Chicago.


ARTICLE IV.

### DUTIES OF DENTIST TO PATIENT—DUTIES OF PATIENT TO DENTIST.

BY A. W. M'CANDLESS, D. D. S., CHICAGO.

Read before the Illinois State Dental Society, May, 1895.

I shall have very little to say on the second part of my subject, as, in some respects, I think the patient better qualifed to speak from that standpoint, hence, in order that a variety of views might be expressed, I asked three of my patients to write on the 'Duties of Patient to Dentist," and will, as a fitting addenda to what I have to say, read you the sentiments of each of these persons on the subject before us.

The first duty of a dentist to his patient is to study his temperament. Different people must be handled differently, and I think it is well that such is the case, because this fact gives us something by way of variety, which relieves what would otherwise be a monotonous humdrum existence.

We have an opportunity to exercise our ingenuity as to the best method to pursue with each individual case presented. We all know what a difference there is in people, and how one person can exhaust our patience and wear us out in a few moments' time, whereas others will give us no worry or fatigue; but unfortunately for us, we cannot select entirely from the latter class, but must take them as they come. Having satisfied ourselves as to the temperament of the individual, the next step is to gain his confidence. This may be successfully done in the case of very nervous people by pretending to accomplish something without causing the least pain; detain the patient but a short time at the first appointment by cleaning the teeth, or at least, doing what you may in that direction without causing distrust, fear or any unpleasant sensation. You will notice such a patient takes the chair with nerves wrought up to a high tension, every muscle rigid, and the person in a frame of mind bordering on distraction. You will observe a little later on, if you follow the suggestion here pointed out, that the muscles begin to relax, fear and distrust presently give way to a feeling that "this isn't going to be so bad after all," then gradually this apprehension grows less and less as your gentle manner convinces him that you appreciate his feelings, until you gain his entire confidence, and a sense of security comes over him that while he is in your hands he will be treated as an animate being that hath flesh and blood, and is capable experiencing pain. If you will pardon me for an illustration of a personal nature, I will mention it here as it is so appropos of the thought presented.

A gentleman of perhaps thirty-eight or forty years of age was introduced to me a few weeks ago by one of my patients, and I could see by the expression of the man's face that he came more to please his friend than because he had any desire to make the acquaintance of a new dentist, as he was fairly well satisfied with his old one. However, he took the chair, I examined his teeth, explained to him what was necessary to be done, but showed no anxiety whatever to secure him for a patient, as I had made up my mind that he was a little doubtful of the advisability of making a change. He told me the name of the dentist who had had the care of his teeth, and I replied. "I have never met the gentleman, but I have heard of him frequently and always in words of praise, as being a fine dentist and a man of large and lucrative practice." This language appeared to please the old gentleman, and he asked for an appointment. When he came again I saw I had a person of very nervous temperament to deal with and one very much afraid of being hurt. I cleaned his teeth, and caused him no pain; At the next sitting I opened up some proximal cavities and filled them with gutta-percha, and explained to him that that was for the purpose of separating his teeth without pain, that the process would require from three to six months, but that in the meantime the gutta-percha fillings would preserve the teeth as well as any filling he could have. I would say parenthetically that I am indebted to Dr. Bonwill for the gutta percha idea. and I think it a splendid one.

I dismissed my patient after keeping him but a short time for his second appointment, and so I have nursed him along until now I have destroyed and removed the pulp of an aching third molar, and have him almost ready for a little bridge, and a few days ago he was kind enough to tell me that he had lived in Chicago for the past twenty years during which time he had never had a dentist that he wanted to tie to, and that when any of his friends would ask him who his dentist was, he would reply, "Oh, no one in particular. I have never had a great deal done to my teeth. But," said he, "I like the way you go at the fellow. You don't go in 'hammer and tongs,' as if you were working on something that had no feeling, and you don't keep a fellow long at a time. As soon as my wife comes home from California I shall bring her in and have you attend to her teeth. She has been going to another town twice a year to her dentist who had the care of her teeth when she lived there, but you are good enough for me, and I think I can do you some good." I mention this particular case simply to show you that it pays to be humane. Leaving the sympathetic side of the question out and for a selfish motive alone it is good policy to be gentle, for your patients will be so grateful that they will inconvenience themselves very often to not only recommend their friends to you but bring them as well.

And right here comes the thought that my subject is incomplete, for I should also have incorporated with it the duties we owe to each other. If there is one thing I dislike in a person more than another it is that characteristic in a man that allows him to say nothing in praise of another in the same calling. It is a mistake for a man to cultivate the idea that in himself alone are all the good points assembled to the exclusion of all the bad. An individual gets in the habit of speaking sneeringly of a brother dentist, or perhaps even contemptuously. It is remarkable how fast this habit will grow, and the more one indulges in it the more opinionated and disagreeable he becomes; and when he arrives at such a stage he makes himself unhappy and miserable, and takes no pleasure or delight in the company of his fellows. On the other hand, let him be charitable, always speaking well of his brothers who conduct themselves in an honorable manner, let him feel that not all the ability, honor and majesty of his profession is wrapped up in himself, but that his peers are all about him and I will show you a pleasant man, a companionable man, and a man who is always willing to receive or impart instruction—in short, a happy man. Habits are easily formed, but not so easily shaken off. Happy then the man who makes up his mind that there are lots of good dentists in Illinois besides himself, and is not afraid to say so. Some men are afraid to speak well of a confrere or competitor, if you choose, lest those sentiments might cause their patients to leave them and try the other; but it is a grave error. The ordinary individual takes with a great deal of allowance what you may say that is derogatory of another dentist, for he makes up his mind at once that were you not engaged in the practice of the same profession you would not say these disagreeable things. On the contrary, you increase your patient's admiration for yourself by being big enough and broad enough to speak in terms of praise of other dentists. Hence, to enlarge your own practice, to place yourself on a high pedestal in the estimation of your patients, to increase the flow of the milk of human kindness in your veins, to make yourself happier, better, truer and nobler, speak well of your brother dentists, and thus more nearly approach the truest type of manhood.

Having gained the confidence of your patient do not lose it by deceiving him. My experience teaches me that it pays to be honest with my patient. There are times when to cause pain is absolutely essential to thoroughness. In such a case I say, "Now I shall hurt you here for a few moments—do you think you can stand it?" By asking that question you appeal to his pride, and he will invariably answer "yes," even though he be but a small child. In that manner you get his help, and it makes your work easier and pleasanter. If you always tell a patient you will *not* hurt him he never believes you, but if you tell him, "now this *will* hurt you," he believes you always; then if you say "I will *not* hurt you this time," he will believe you; he relaxes the muscles; he does not resist you, consequently the work is made easier for both. I would not have you infer from what I have said that you should humor a patient beyond a reasonable limit. To be sure, with little children our patience is taxed oftimes almost to the point of exhaustion, but in the case of adults I do not think it necessary to go to such extremities. If they will not be reasoned with, if they cannot be persuaded that you are getting no particular pleasure out of their pain, if they absolutely refuse to help you so that you may be enabled to do what is best for them, there your duty ends. It is a waste of time and energy and vitality to try to benefit such people. Then it is time to show them politely but firmly that if you cannot have their co-operation you will stop right there and then. Many persons are by this last resort brought for the first time to realize how much they themselves have been to blame, and will be ashamed of their conduct. Therefore, one chief duty of a dentist to his patient is firmness.

It is the duty of the dentist to use all the aids that advanced thought and investigation have given us to relieve pain. It is your duty to have everything about your office as tasty, as complete and as attractive as the situation and the circumstances will permit, to have every approved appliance to lessen the labor, to have every disagreeable feature as much in the background as possible. Other things being equal, the dentist who has a clean, well-appointed, well-kept complete office, has a big advantage over the fellow who has not. It is the duty of every dentist who practices in a town large enough to have a water supply, to have a fountain spittoon and a saliva ejector. It is his duty, in short, to make the operating room as much unlike the chamber of horrors as it is possible. It is his duty to be successful. My father gave me as his parting advice, when I left home a good many years ago, this motto—"Remember there's nothing succeeds like success." Therefore make a big bluff at being successful anyway. People like to do business with a successful man—there is something contagious about it.

To keep your practice well in hand and to give your patients the best possible attention send for them at least twice a year. When I finish with a patient I say; "Now, if you wish me to have the care of your teeth I shall want to see you every six months, then I can take the cavities before they encroach upon the pulp, and, consequently, you will save yourself expense and your teeth will be kept in better condition, which will be much more satisfactory to you and to me." This is something your patients will appreciate if they take a proper pride in keeping their mouths in a nice healthy condition.

It is the dentist's duty to charge well for his services? a duty, because good fees impel good services. If I make a crown for ten dollars, for instance, and I find I have missed it a little on the articulation, or the contour is not just what it ought to be, or I find the root has not been sufficiently trimmed, how natural it would be for me te say, "Oh, well, I'm only getting ten dollars for this anyway, and it is good enough;" but suppose I get twenty-five dollars for a crown, how much more apt am I to say, "Oh, ho! this will not do! Here is a patient paying me full price for this crown and he is entitled to the very best that I know how to do for him." You might say that the truly conscientious man would do his very best on a crown whether he received five dollars or twenty-five; but, gentlemen, it is not human nature. People do not expect to get seal skins for the price of plush.

Therefore, gentlemen, I say one of your duties to your patients, and a very important one, is to charge well for what you do, because good prices impel good work and bring good people.

Another duty a dentist owes his patient is to attend and belong to the State Dental Society, and as many others as he may be able to attend, in order that he may keep abreast of the times to give his patients the benefit of all that is latest and best.

As I write, this subject grows on me so that I find enough might be said to fill a good sized volume, but do not be frightened, I shall close by saying there are many other duties that we owe our patients indirectly. One is to conduct ourselves in an ethical and upright manner, another is, "Be sure you are a member of the Dental Protective Association," for by so doing you find yourself in good society; another, subscribe for all the good dental journals. And lastly, do your very best at anything and everything you undertake. There lies the secret of permanent success, and by doing your best, "your best will better grow," and in due time ye shall reap your reward.—*Southern Dental Journal and Luminary*.

